# Variations in mood states among Brazilian air force pilots: a crossover analysis between operational and aerobatic flight missions

**DOI:** 10.3389/fphys.2025.1643371

**Published:** 2025-11-24

**Authors:** Wilian de Jesus Santana, Gilberto Pires Pivetta, Carlos Eduardo Rosa Da Silva, Fábio Angioluci Diniz Campos, Paulo Farinatti, Gustavo Almeida, Adriano da Silva Verame, Regina Brandão, Aylton J. Figueira Junior

**Affiliations:** 1 São Judas Tadeu University, São Paulo, Brazil; 2 Piaget University Center - UNIPIAGET, Suzano, Brazil; 3 Air Force University, Rio de Janeiro, Brazil; 4 Laboratório de Atividade Física e Promoção da Saúde, Universidade do Estado do Rio de Janeiro, Rio de Janeiro, Brazil

**Keywords:** mood states, aerobatic flight, military aviation, affective monitoring, occupational psychophysiology

## Abstract

**Background:**

Aerobatic flight maneuvers expose military pilots to a confluence of physiological and psychological stressors—including sustained G-forces, vestibular disorientation, and neuromuscular fatigue—that may acutely modulate emotional states and compromise cognitive performance. However, the differential psychometric impact of routine operational flights versus high-intensity air demonstrations remains poorly characterized.

**Purpose:**

To examine and compare mood state fluctuations elicited by distinct aerial mission profiles—operational maintenance (OM) flights and aerobatic demonstrations (AD)—within a specialized military pilot cohort.

**Methods:**

In this within-subject, crossover experimental design, nine elite male aviators (mean age: 32.1 ± 1.8 years) from the Brazilian Air Force Air Demonstration Squadron (EDA), each with >1,000 cumulative flight hours, were assessed across two mission contexts. Mood states were measured pre- and post-flight using the Brunel Mood Scale (BRUMS), which evaluates six psychological dimensions: tension, depression, anger, vigor, fatigue, and confusion. Paired comparisons were conducted using the Wilcoxon signed-rank test (α = 0.05).

**Results:**

OM flights were associated with a statistically significant increase in depression scores post-flight (p = 0.031), alongside a non-significant trend toward decreased tension (p = 0.098). Other subscales showed no statistically significant variation, though intra-individual variability was observed.

**Conclusion:**

Emotional reactivity among high-performance military pilots varies according to mission typology. Incorporating continuous affective monitoring and routine psychological assessments may bolster both operational readiness and mental health preservation in high-demand aviation environments.

## 1 Introduction

Military flight operations impose exceptional cognitive, physiological, and emotional demands on aircrew. Pilots are subject to multifaceted stressors, including gravitational acceleration (G-forces), vibrational stimuli, spatial disorientation, and fatigue, which disrupt homeostatic balance and may impair mood regulation, executive function, and operational performance (Convertino, 1999; Sauvet et al., 2009; Borghini et al., 2014).

Within the domain of aerobatic aviation, these stressors are further exacerbated by the demands of precision maneuvering, heightened sensory processing, and split-second decision-making. Pilots navigating these high-stakes conditions must contend with dynamic autonomic responses and psychophysiological strain that may predispose them to affective dysregulation and neurocognitive fatigue (Afraimovich et al., 2011; Gül and Salmanoglu, 2012).

Mood states, defined as transient yet pervasive affective dispositions, are known to fluctuate in response to situational, environmental, and internal cues. These fluctuations may serve as early indicators of psychological strain or resilience. The Brunel Mood Scale (BRUMS) has emerged as a robust, sensitive psychometric tool capable of capturing subtle affective shifts in high-performance populations, particularly in sport and military contexts (Lane and Terry, 2000; Terry et al., 2003).

Unlike previously studied populations, the present investigation targets elite military aviators, a highly trained and operationally constrained cohort routinely subjected to extreme flight conditions. To the best of our knowledge, no prior studies have systematically examined mood state dynamics in response to distinct aerial mission types within this specific population.

Air Force pilots represent a professional group particularly vulnerable to the development of mental fatigue, a condition that detrimentally affects attentional control, situational awareness, and decision-making efficiency (Ferreira et al., 2025). These vulnerabilities underscore the necessity of stringent selection, training, and psychophysiological screening protocols to mitigate operational risk (Socha et al., 2020).

This study offers novel empirical insights into the affective responses of military pilots, thereby addressing a critical gap in the literature on aviation psychology. Its findings hold translational potential for developing evidence-based preventive strategies and intervention programs aimed at sustaining psychological resilience and mission-readiness in high-performance flight operations. The primary objective was to compare mood state alterations elicited by different types of aerial missions—operational maintenance (OM) and aerobatic demonstrations (AD)—within the Brazilian Air Force’s Air Demonstration Squadron (ADS).

## 2 Materials and methods

### 2.1 Study design

A within-subject, crossover experimental design was employed. Each participant underwent two distinct flight conditions: an operational maintenance (OM) flight and an aerobatic demonstration (AD) flight. Experimental sessions were conducted under standardized methodological protocols to ensure consistency across conditions. The order of exposure was fixed, with all participants first completing the OM flight followed by the AD flight, allowing for temporal control and minimization of carryover effects. The flights were carried out with differences of at least 7 days between them, thus reducing possible order effects, such as the accumulation of fatigue or anticipatory stress.

### 2.2 Subjects

The study sample comprised nine male pilots (mean age: 32.1 ± 1.8 years), all active-duty members of the Brazilian Air Force’s elite Air Demonstration Squadron. Inclusion criteria required clinical clearance for flight activity and a minimum threshold of aerial experience; participants averaged 1,640 ± 1,058 cumulative flight hours. Exclusion criteria encompassed current use of neurostimulant substances or any temporary medical restriction from flight duties. Although the numerical sample size is limited, it represents approximately 60% of the entire operational squadron which had 15 pilots active, thus ensuring robust representativeness and high ecological validity for this specialized context.

### 2.3 Procedures

**FIGURE 1:**
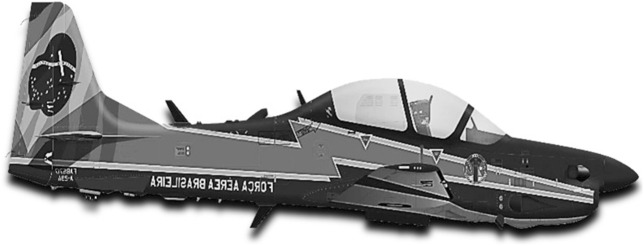
A-29 aircraft–super tucano–FAB. Fonte: https://historiadafab.rudnei.cunha.nom.br/2020/12/28/embraer-emb-314-a-29-super-tucano/.

All experimental procedures were conducted using the A-29 Super Tucano (EMB-314), a light attack turboprop aircraft designed for advanced pilot training (Figure 1), equipped with modern avionics and weapons systems. The aircraft is capable of sustained cruise speeds up to 590 km/h and is operationally employed in both tactical and instructional missions by the Brazilian Air Force.

Data collection occurred across three separate assessment session, as follows:

- Anthropometric evaluation and cardiorespiratory fitness: Anthropometric assessment and estimation of maximal oxygen uptake (VO_2_max) via the Yo-Yo Intermittent Recovery Test, a validated field-based proxy for cardiorespiratory fitness;
- Operational Maintenance (OM) mission: Operational maintenance flights are characterized by lower technical and cognitive complexity, as they primarily consist of weekly aircraft checks. In this context, the pilot performs maneuvers in isolation, with greater time available for pre-mission planning and reduced demands for simultaneous coordination;
- Aerobatic Demonstration (AD) mission: In contrast, air demonstration flights require substantially more rigorous preparation and higher levels of training. During these missions, the pilot operates in formation with six other aircraft, necessitating precise real-time synchronization. The maneuvers executed involve a high degree of risk, including high-speed crossovers, abrupt acceleration changes, and attitude inversions, thereby imposing greater psychophysiological load and demanding rapid decision-making under extreme conditions.

All sessions adhered strictly to standardized methodological protocols to ensure procedural uniformity and control for environmental and operational variability. The within-subject experimental sequence was structured to allow each participant to serve as his own control, thereby enhancing the reliability of intra-individual comparisons and minimizing between-subject confounders.

Subsequent sections provide detailed descriptions of the psychometric instruments, physiological parameters, and statistical methods employed in this investigation.

In the second experimental session, participants completed the Operational Maintenance (OM) flight, with an average duration of 50 min, conducted at varying times throughout the day (morning and afternoon). Pilots were instructed to maintain their habitual routines, including unrestricted intake of food, beverages, and prescribed medications. All flight maneuvers, both in the OM and Aerobatic Demonstration (AD) conditions, were executed according to standardized protocols pre-established by squadron command.

During the AD mission, seven aircraft flew simultaneously in synchronized formation, performing complex aerobatic sequences involving both group and solo maneuvers. The OM flight served as the baseline (control) condition, while the AD mission represented the experimental (intervention) condition. Mood states were assessed immediately pre- and post-flight using the Brunel Mood Scale (BRUMS). Both mission types lasted an average of 50 min and were distributed across morning and afternoon time slots. The OM and AD flights were carried out in isolation, with weeks between them, suggesting the risk of order interference in the relationship between OM and AD flights, such as accumulation of fatigue or anticipatory stress.

### 2.4 Instruments

#### 2.4.1 Psychometric instrument

The Brunel Mood Scale (BRUMS) is a 24-item, self-administered questionnaire designed to assess six mood dimensions: tension, depression, anger, vigor, fatigue, and confusion. Participants rate their affective state in response to the prompt “How do you feel right now?” using a five-point Likert scale ranging from 0 (not at all) to 4 (extremely) (McNair et al., 1971). The BRUMS has demonstrated high validity and reliability for use in sports and military populations. The Brazilian Portuguese version utilized in this study was validated by Rohlfs et al. (2008). The instrument was administered immediately before and after each aerial mission to capture acute mood fluctuations.

### 2.5 Statistical analysis

An *a priori* sampling power analysis was conducted in the G*Power 3.1.9.4 software to determine the minimum sample size required. A t-test was considered for dependent measures (sample pair), with moderate effect size (dz = 0.5), significance level of 5% (α = 0.05), statistical power of 80% (1 – β = 0.80), and a one-tailed hypothesis. The calculation indicated the need for 27 participants to achieve adequate power. However, due to the operational constraints of the study, the final sample was composed of nine elite pilots of the Brazilian Air Force, ensuring high ecological validity, but with reduced statistical power to detect effects of moderate magnitude. After data collection, a normality test was performed, which indicated a non-normal distribution of the data. Therefore, a non-parametric approach was adopted, and comparisons between pre- and post-flight conditions were conducted using the Wilcoxon signed-rank test.

Inferential analyses were performed using the Wilcoxon signed-rank test for paired samples, appropriate for small samples and non-parametric distributions. For each BRUMS subscale, descriptive statistics (mean, standard error, standard deviation, minimum, maximum, and quartiles) were computed. Additionally, a percentage change (Δ%) between pre- and post-flight predictions was calculated from the recommendation = (post-flight - pre-flight)/pre-flight x 100 to describe the magnitude and direction of change.

Effect sizes were determined using the formula r = Z/√n and interpreted according to Cohen’s criteria: small (r ≥ 0.10), moderate (r ≥ 0.30), and large (r ≥ 0.50) (Cohen, 1988). Analyses were conducted in IBM SPSS Statistics 25.0 (IBM Corp., Armonk, NY), and visual representations were created using GraphPad Prism 8.0.2 (GraphPad Software, San Diego, CA). Statistical significance was set at p < 0.05.

### 2.6 Ethics statement

The studies involving humans were approved by CNS Resolution No. 466/12, in accordance with ethical principles standardized by São Judas University Ethics Committee N°: 5.786.099. The studies were conducted in accordance with the local legislation and institutional requirements. The participants provided their written informed consent to participate in this study. Written informed consent was obtained from the individual(s) for the publication of any potentially identifiable images or data included in this article.

## 3 Results

A total of 15 participants were initially evaluated for eligibility. Six were excluded because they did not meet the inclusion criteria, specifically lack of clinical clearance for flight activity, or because they were unavailable due to operational assignment at other bases. In this way, nine drivers made up the final sample. The recruitment process and execution of the experimental protocol lasted for 24 months, with all collections completed by November 2023.

The descriptive characteristics of the sample are presented in Table 1. Discrete differences were observed in anthropometric variables, such as body mass and fat percentage. The in-flight experience was controlled according to the aircraft on which the collections took place, although some participants had additional history in other types of aircraft.

**TABLE 1:** Sample demographic description in Brazilian air force pilots.

Sample	09 pilots
Age (years)	32 ± 3
Body Mass (kg)	73,7 ± 8,1
Height (cm)	178 ± 0,7
% Fat	17,4 ± 4,0
VO2_max_ (mL.kg.min^-1^)	41,6 ± 3,0
Flight Experience (Hours)	1,640 ± 1,058

**FIGURE 2:**
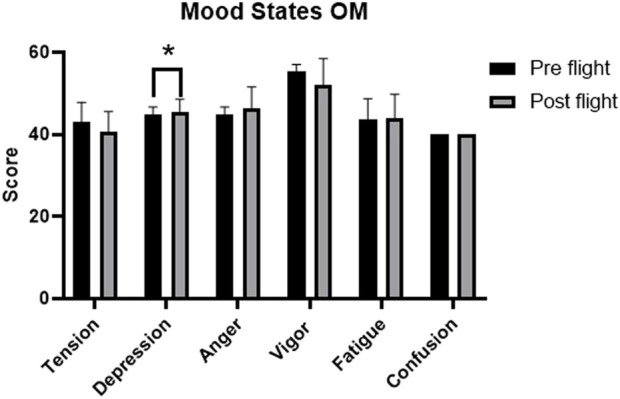
Comparison chart of the mood states of the OM flight. Note: Figure 2 presents the mean scores of mood states before (pre-flight) and after (post-flight) operational maintenance (OM), with error bars representing the standard deviation. The only statistically significant difference was observed in the “Depression” dimension (p < 0.05), highlighted with the asterisk symbol. The other variables did not present significant differences between the moments evaluated, although small variations can be observed in the dimensions of tension, vigor and fatigue.

Mood states during the operational maintenance flight are described in Table 2. There was a statistically significant increase in the depression subscale (p = 0.031) (Figure 2), with moderate effect size (r = 0.33). The strain subscale showed a downward trend, which was not significant (p = 0.098), but accompanied by a large effect size (r = 0.55).

**TABLE 2:** Comparison of OM pre and post-flight mood states of Brazilian air force pilots.

Mood states / moments	N	Mean	Standard error	Minimal	Maximum	Percentis			Δ% of the mean	Z	p value	r (Effect size)	Effect size
						25th	50th (median)	75th					
Tension_before_OM	9	43,0	4,8	37,0	48,0	37,0	44,0	48,0	−5%	−1,656	0,098	−0.55	Big
Tension_after_ OM	9	40,7	4,8	37,0	48,0	37,0	37,0	46,0					
Depression_before_ OM	9	44,9	1,8	44,0	48,0	44,0	44,0	46,0	1%	−1,000	0,031*	−0.33	Moderate
Depression_ after _ OM	9	45,4	3,1	44,0	53,0	44,0	44,0	46,0					
Anger_ before_ OM	9	44,9	1,8	44,0	48,0	44,0	44,0	46,0	3%	−0,816	0,414	−0.27	Small
Anger _ after_ OM	9	46,2	5,3	44,0	60,0	44,0	44,0	46,0					
Vigor_ before_ OM	9	55,2	1,8	53,0	58,0	53,0	56,0	56,0	−6%	−1,194	0,233	−0.39	Moderate
Vigor_after_ OM	9	52,1	6,3	44,0	58,0	44,0	56,0	57,0					
Fatigue_before_ OM	9	43,7	5,0	39,0	54,0	39,0	42,0	46,5	1%	−0,333	0,739	−0.11	Small
Fatigue_after_ OM	9	44,0	5,8	39,0	57,0	39,0	42,0	46,5					
Confusion_before_ OM	9	40,0	0,0	40,0	40,0	40,0	40,0	40,0	0%	0,000	1,000	0	Null
Confusion_after_ OM	9	40,0	0,0	40,0	40,0	40,0	40,0	40,0					

Comparison of the scores of psychometric variables before (pre) and after (post) the intervention in the OM, group (n = 9). The data are presented as mean, standard deviation (Deviation Error), minimum and maximum values, and 25th, 50th (median) and 75th percentiles. The percentage variation (Δ%) of the mean was included only as a descriptive reference. Statistical analysis was performed with the Wilcoxon test for paired samples, appropriate for non-parametric data. The Z value represents the test statistic, and the p value indicates the level of significance. The effect size (r) was calculated with the formula r = Z/√n, as recommended for non-parametric data. The interpretation of the effect follows Cohen’s criteria (Cohen, 1988): small (r ≥ 0.10), moderate (r ≥ 0.30) and large (r ≥ 0.50). * = Values of p < 0.05 were statistically considered.

**FIGURE 3:**
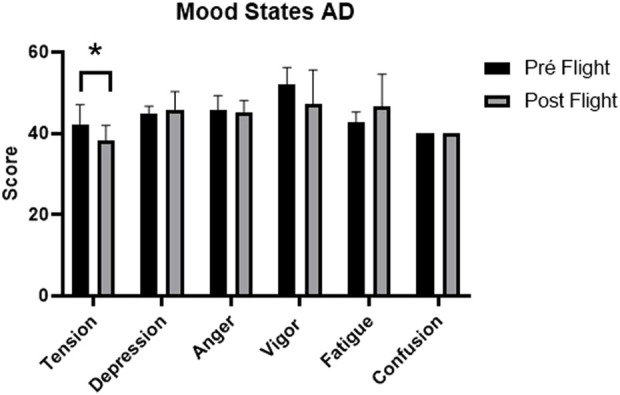
Comparison chart of AD flight mood states. Note: Figure 3 shows the mean scores of the mood states assessed before (pre-flight) and after (post-flight) of the mission in the AD group, with error bars representing the standard deviation. The only statistically significant difference was observed in the “Tension” dimension (p < 0.05), indicated by the asterisk. The other variables depression, anger, vigor, fatigue and confusion did not present significant differences between the moments evaluated, although slight oscillations can be seen. These data complement the descriptive and inferential statistical analysis presented in the corresponding tables.

**FIGURE 4:**
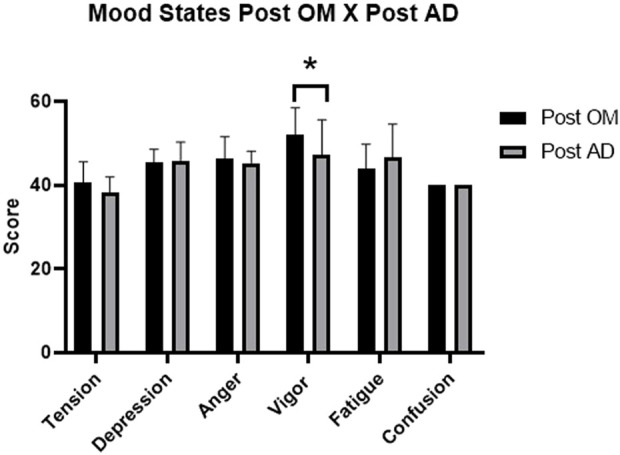
Graph of the comparison of the post-flight mood states of OM X AD. Note: OM = Operational Maintenance; AD = Aerial Demonstration. The bars represent the mean scores and standard deviation of mood states at the post-flight moment of each mission. * - indicates a statistically significant difference between the groups (p < 0.05).

In the condition of aerial demonstration, the data referring to mood states are shown in Table 3. A statistically significant reduction in tension (p = 0.041) was observed, associated with a very large effect size (r = 0.68). The other dimensions (Figure 3), including depression, anger, vigor, fatigue, and confusion, were not statistically significant, but some showed moderate effect sizes. The comparison between the conditions of operational maintenance and aerial demonstration is shown in Figure 4. The tension and confusion scores remained similar between the two situations. The depression, anger, and fatigue subscales also did not differ significantly between flight types.

**TABLE 3:** Comparison of AD pre- and post-flight Mood States of Brazilian Air Force Pilots.

Mood states / moments	N	Mean	Standard error	Minimal	Maximum	Percentis			Δ% of the mean	Z	p value	r (Effect size)	Effect size
						25th	50th (Medium)	75th					
Tension_before_AD	9	42,3	4,8	37,0	48,0	37,0	41,0	48,0	−10%	−2,041	0,041*	−0.68	Big
Tension_after_ AD	9	38,2	3,7	37,0	48,0	37,0	37,0	37,0		−1,000			
Depression_before_ AD	9	44,9	1,8	44,0	48,0	44,0	44,0	46,0	2%	−1,000	0,317	−0.33	Moderate
Depression_after_ AD	9	45,9	4,4	44,0	57,0	44,0	44,0	46,0		−1,625			
Anger_before _ AD	9	45,8	3,5	44,0	52,0	44,0	44,0	48,0	¨-1%	−1,404	0,317	−0.47	Moderate
Anger _after _ AD	9	45,3	2,8	44,0	52,0	44,0	44,0	46,0		0,000			
Vigor_before _ AD	9	52,0	4,2	47,0	56,0	47,0	53,0	56,0	−9%	−0,962	0,104	−0.32	Moderate
Vigor_after _ AD	9	47,3	8,3	35,0	56,0	39,5	47,0	56,0		−0,577			
Fatigue_before _ AD	9	42,7	2,5	39,0	45,0	40,5	42,0	45,0	9%	−0,447	0,160	−0.15	Small
Fatigue_after _AD	9	46,6	7,9	39,0	65,0	41,0	48,0	48,0		−2,226			
Confusion_before _AD	9	40,0	0,0	40,0	40,0	40,0	40,0	40,0	0%	−1,404	1,000	−0.47	Moderate
Confusion_after _AD	9	40,0	0,0	40,0	40,0	40,0	40,0	40,0		0,000			

Comparison of psychometric scores before (pre) and after (post) the intervention in the AD, group (n = 9). The data are presented as mean, standard deviation (Deviation Error), minimum and maximum values, and 25°, 50° (median) and 75° percentiles. The percentage change in the means (Δ%) is an additional descriptive measure. The Wilcoxon test for paired samples was used for inferential analysis, appropriate for small samples and non-parametric data. The Z statistic and the p-value are presented for each comparison. The effect size (r) was calculated using the formula r = Z/√n, where n is the number of participants (n = 9). The interpretation of the effect size follows Cohen’s criteria (Cohen, 1988): small (r ≥ 0.10), moderate (r ≥ 0.30) and large (r ≥ 0.50). Results with p < 0.05 were considered statistically significant.

Vigor showed a significant reduction after aerial demonstration flights compared to operational maintenance flights (p = 0.026). This finding was the only statistically confirmed contrast in the direct comparison between the conditions. In general, the operational maintenance and air demonstration flights did not result in significant differences for most of the mood dimensions. However, the combination of results showed decreased vigor, a tendency to increase fatigue and increased depression in both conditions (Lane et al., 2004).

The pre- and post-flight analysis of operational maintenance indicated a downward trend in the stress scores, without statistical significance (p = 0.098), but accompanied by a large effect size (r = 0.55). This finding suggests that, even in the absence of statistical robustness, there is a possible psychological relevance, considering that lower levels of tension are associated with greater mental health and better performance in cognitive and physical tasks (Morgan, 1985; Morgan et al., 1987).

The other subscales of the BRUMS (anger, vigor, fatigue, and confusion) showed no significant changes, exhibiting small effect sizes. Even so, the descriptive analysis revealed slight variations, with emphasis on the depression dimension, which showed a consistent increase. This stability in multiple dimensions, accompanied by an increase in depression, points to a pattern of psychophysiological response that deserves attention.

The literature maintains that depression is the most relevant dimension in the structure of mood, due to its demotivating nature and the negative impact on other affective variables (Lane et al., 2005). The combination of high depression and anger has been described as detrimental to performance, while anger alone, in individuals without depressive mood, may function as a motivating factor (Lane et al., 2005). This situation may explain the absence of a positive compensatory effect of rabies in the present study.

Reduced stamina and increased fatigue are considered common responses to intense exertion. However, when accompanied by an increase in depression levels, such findings may characterize a psychophysiological maladaptation to the workload (Lane et al., 2004). In the context of military aviation, this triad can represent operational risk, as it directly impacts the pilot’s motivation, readiness, and decision-making capacity.

Previous studies with athletic populations have shown a dose-dependent relationship between training load and mood swings, where increased volume or intensity was associated with affective disturbances and reduced load resulted in improved mood (Bouget et al., 2006). This dynamic can be extrapolated to aerial activity, in which operational overload similar to aerial demonstration flight could induce negative psychological responses.

Factors such as previous experiences, expectations, coping strategies, and environmental pressures play a critical role in modulating mood in pilots (Vieira et al., 2010). Thus, the interaction between individual and environmental variables needs to be considered when interpreting the results of the present study, especially in scenarios of high operational complexity.

In the aerial demonstration condition, the results showed a statistically significant reduction in tension scores (p = 0.041), with a very large effect size (r = 0.68). This response suggests that the execution of aerobatic maneuvers, although requiring high cognitive and motor load, can induce a compensatory reduction in tension after the flight. The other subscales did not reach statistical significance, but presented moderate effect sizes, which suggests relevant clinical trends. This pattern indicates that psychological changes may not be robustly expressed at the statistical level, but they still impact the subjective experience of pilots.

## 4 Discussion

The present investigation aimed to understand the affective fluctuations in the face of different air mission profiles. The comparison between operational maintenance flights and aerobatic demonstration allowed the identification of distinct response patterns, with special emphasis on tension and vigor as the most sensitive dimensions. The operational maintenance flight, characterized by less complexity, contrasts with the aerial demonstration flight, in which multiple aircraft perform synchronized maneuvers with high precision. This structural difference was reflected in the findings, since only the strain showed a significant drop after the aerobatic mission, while the vigor declined consistently, but not statistically significant. These results underscore the role of task intensity and mission complexity in modulating pilots’ psychophysiological and emotional responses, even among highly trained individuals (Hart and Staveland, 1988; Cardoso and Gontijo, 2012).

The decline in vigor accompanied by increased fatigue in the acrobatic condition, approximately 6% on average, points to a substantial psychophysiological load. Flight simulations demonstrate that increased cognitive load triggers sympathetic activation and reduces heart rate variability (indicative of parasympathetic deactivation) (Koskelo et al., 2024), a phenomenon compatible with that observed in real/aerobatic flight. Conditions in which there is an alteration in the G-load highlight that fine motor precision suffers under high mechanical and vestibular stress (Guardiera, Schneider, Noppe and Strüder, 2008), reinforcing that such tasks demand sustained attention, emotional regulation and fine motor coordination.

The error bars suggest marked inter-individual variability in the reaction to operational demands. This heterogeneity may be associated not only with the level of physical fitness of the pilots, but also with the degree of emotional resilience and individual coping strategies. Greater resilience correlates with better autonomic flexibility in stressful situations (An et al., 2020), while personality differences, such as high negative affectivity or avoidant coping styles, modify both physiological activation and subjective perception of workload (Grassmann et al., 2017).

The results corroborate the hypothesis that more complex missions induce greater psychophysiological cost, as demonstrated by heart rate increases and decreased autonomic variability in events of high urgency and low familiarity with the flight scenario (Peng et al., 2025), and correlations between elevated cognitive load and HRV changes in more demanding maneuvers (Zhou et al., 2024; Wang et al., 2024). The significant reduction in vigor after aerobatic flights confirms the impact of operational complexity on the affective state, reinforcing vigor as a sensitive marker of pilot adaptation (Terry, 1995). This finding is in line with the literature that relates changes in vigor and fatigue to autonomic modulation. Changes in sympathetic-vagal balance, associated with increased perception of effort, may explain the drop in post-mission vigor. Such a response has direct implications for operational readiness and flight safety (Rangel, 2022; Parry-Williams and Sharma, 2020).

Flight experience showed no significant association with BRUMS dimensions in this study, suggesting that cumulative time in service may not be sufficient to mitigate the acute psychological effects of high-demand missions. This contradicts part of the literature that attributes experience to a protective role in the emotional stability of pilots (Temme et al., 2023). Despite this, the findings indicate that emotional regulation mechanisms preserve psychological resilience, especially in tension control, even in highly demanding contexts. This regulation, however, does not eliminate the internal cost reflected in the decrease in vigor.

Continuous mood monitoring in military pilots emerges as a preventive strategy. Discrete alterations, even without statistical significance, can signal progressive emotional exhaustion and serve as a warning for occupational health managers. Repeating missions can contribute to greater emotional control, but it does not eliminate the physiological cost of adaptation. Recent research points to dissociation between affective stability and internal exhaustion, which reinforces the need for integrated psychophysiological assessments (Norrholm et al., 2023).

The literature on high-performance sports shows important parallels. Altered mood states negatively impact motor performance, coordination, attention, and mental health (Bouget et al., 2006; Covassin and Pero, 2004; Lane et al., 2004; Peluso, 2003; Rohlfs et al., 2004). These mechanisms can be extrapolated to aviation, where motor precision and emotional regulation are decisive for mission safety.

The results of the present study reinforce the importance of vigor and tension as key indicators in the monitoring of pilots. Their sensitivity suggests that they should be incorporated into operational monitoring protocols, functioning as affective biomarkers of the psychophysiological state Since previous studies have highlighted the important role of emotional management in cognitive interference during performance, suggesting that emotions can alter the state of attention and judgment (Forgas, 1995; McCartney et al., 2013; Stranger et al., 2018). The integration of affective measures with physiological assessments, such as heart rate variability and stress biomarkers, represents a promising path to broaden the understanding of adaptation in aeronautical scenarios. This multidimensional approach can guide interventions aimed at maintaining health and performance in military pilots.

Finally, the discussion of the findings shows that military aviation, due to its complexity and psychophysiological demands, demands not only technical preparation, but also continuous psychological monitoring. The data obtained provide initial evidence to support prevention and support programs in occupational health, strengthening safety and performance in aerial activity.

## 5 Study limitations

Despite the methodological rigor and operational relevance of the sample composed of elite pilots of the Air Demonstration Squadron of the Brazilian Air Force, some limitations must be recognized. First, the small sample size (n = 9) severely constrains statistical power and increases the likelihood of Type I and II errors, as predicted by *a priori* power analysis. However, this is a sample that represents about 60% of the active population of the unit, which reinforces the relevance and applicability of the findings. Second, the fixed sequence of exposure, in which operational maintenance flights have always preceded air demonstration flights, raises the possibility of order effects, such as accumulation of fatigue or anticipatory stress. However, the evaluations were performed with a minimum interval of 1 week between sessions, which substantially reduces the probability of bias due to this factor.

Finally, the specificity of the sample is highlighted. While the inclusion of elite demonstration pilots increases the ecological validity and operational relevance of the results, it limits generalization to other populations of military or civilian aviators. Therefore, the findings should be interpreted as characteristic of this highly specialized profile, rather than extrapolated indiscriminately.

## 6 Conclusion

In summary, this study investigated the acute affective effects of different types of air missions on elite military pilots, identifying that operational maintenance and air demonstration flights modulate mood in a different way. Both mission profiles were associated with reductions in negative affective indicators, but differed in terms of impact on vigor: operational maintenance flights induced an average drop of 6%, while air demonstration flights caused a decrease of 9%, reflecting a higher psychophysiological cost.

These results show that the characteristics of the mission directly shape the emotional response, with routine flights favoring positive affective recovery, while aerobatic missions impose a greater psychophysiological load, expressed in emotional exhaustion. The consistent pattern observed in the post-mission vigor reinforces the need for specific recovery strategies, adapted to the profile of the operation. In this context, the BRUMS scale emerges as a practical and sensitive tool for psychological screening, allowing the monitoring of affective readiness, the early detection of emotional overload, and the implementation of individualized preventive interventions in training programs and operational routine.

Finally, the findings reinforce that high-performance military aviation requires not only technical preparation, but also systematic psychological monitoring. Future research should prioritize larger and more diversified samples, incorporate physiological biomarkers (such as cortisol), and adopt longitudinal designs, in order to deepen the understanding of the psychophysiological mechanisms of adaptation in scenarios of high operational complexity.

## Data Availability

The original contributions presented in the study are included in the article/Supplementary Material, further inquiries can be directed to the corresponding author.
